# Mitochondria and cytochrome components released into the plasma of severe COVID-19 and ICU acute respiratory distress syndrome patients

**DOI:** 10.1186/s12014-023-09394-0

**Published:** 2023-04-08

**Authors:** Zhuo Zhen Chen, Lloyd Johnson, Uriel Trahtemberg, Andrew Baker, Saaimatul Huq, Jaimie Dufresne, Peter Bowden, Ming Miao, Ja-An Ho, Cheng-Chih Hsu, Claudia C. dos Santos, John G. Marshall

**Affiliations:** 1 Department of Chemistry and Biology, Faculty of Science, Toronto Metropolitan, University, 350 Victoria Street, Toronto, ON Canada; 2 St. Michael’s Hospital, Keenan Research Centre for Biomedical Science, Toronto, Canada; 3 YYZ Pharmatech Inc, Toronto, Canada; 4 Department of Chemistry, National Taiwan University, Taipei, Taiwan; 5 Integrated BioBank of Luxembourg, Luxembourg Institute of Health, 6 R. Nicolas-Ernest Barblé, Luxembourg, Luxembourg

## Abstract

**Introduction:**

Proteomic analysis of human plasma by LC–ESI–MS/MS has discovered a limited number of new cellular protein biomarkers that may be confirmed by independent biochemical methods. Analysis of COVID-19 plasma has indicated the re-purposing of known biomarkers that might be used as prognostic markers of COVID-19 infection. However, multiple molecular approaches have previously indicated that the SARS-COV2 infection cycle is linked to the biology of mitochondria and that the response to infections may involve the action of heme containing oxidative enzymes.

**Methods:**

Human plasma from COVID-19 and ICU-ARDS was analyzed by classical analytical biochemistry techniques and classical frequency-based statistical approaches to look for prognostic markers of severe COVID-19 lung damage. Plasma proteins from COVID-19 and ICU-ARDS were identified and enumerated versus the controls of normal human plasma (NHP) by LC–ESI–MS/MS. The observation frequency of proteins detected in COVID-19 and ICU-ARDS patients were compared to normal human plasma, alongside random and noise MS/MS spectra controls, using the Chi Square (χ^2^) distribution.

**Results:**

PCR showed the presence of MT-ND1 DNA in the plasma of COVID-19, ICU-ARDS, as well as normal human plasma. Mitochondrial proteins such as MRPL, L2HGDH, ATP, CYB, CYTB, CYP, NDUF and others, were increased in COVID-19 and ICU-ARDS plasma. The apparent activity of the cytochrome components were tested alongside NHP by dot blotting on PVDF against a purified cytochrome c standard preparation for H_2_O_2_ dependent reaction with luminol as measured by enhanced chemiluminescence (ECL) that showed increased activity in COVID-19 and ICU-ARDS patients.

**Discussion:**

The results from PCR, LC–ESI–MS/MS of tryptic peptides, and cytochrome ECL assays confirmed that mitochondrial components were present in the plasma, in agreement with the established central role of the mitochondria in SARS-COV-2 biology. The cytochrome activity assay showed that there was the equivalent of at least nanogram amounts of cytochrome(s) in the plasma sample that should be clearly detectable by LC–ESI–MS/MS. The release of the luminol oxidase activity from cells into plasma forms the basis of a simple and rapid test for the severity of cell damage and lung injury in COVID-19 infection and ICU-ARDS.

**Graphical Abstract:**

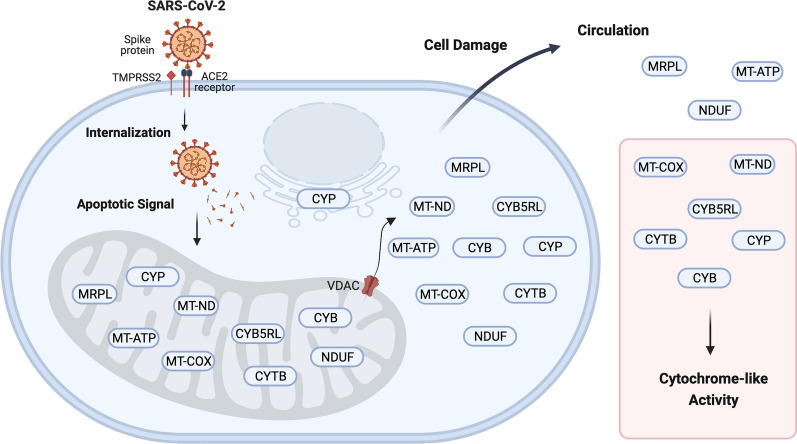

## Introduction

Proteomic analysis of COVID-19 patient plasma has previously indicated that AZGP1, B2M, CRP, HP, HPR, ORM, RBP4, and some SAA may be repurposed as biomarkers of COVID-19 [1–11]. Infection with SARS-CoV-2 resulting in COVID-19 may have some symptoms similar to patients experiencing Acute Respiratory Distress Syndrome (ARDS) [12]. The SARS-CoV-2 spike protein may be cleaved by a protease presumed to be TMPRSS2 to gain entry to the cell through the ACE2 receptors [13]. Subsequently, the spike protein may act as a trigger of apoptosis via a mitochondrial pathway [14]. The release of mitochondrial cytochrome is known to trigger apoptosis of cells [15]. Viral infection resulted in major re-arrangements of cellular compartments including mitochondrial perinuclear clustering, association with the ER and Golgi apparatus, and the fission of mitochondria with the release of reactive oxygen species [16]. The mitochondria may play a central role in the physical interactions of SARS-CoV-2 during the viral replication cycle [17]. Expression of mitochondrial porin forms a channel for the release of macromolecules from the mitochondria organelle into the cytoplasm of COVID-19 patients and thereby acts as a cell death regulator [18]. Viral infection may permit the escape of large molecules like mitochondrial DNA and proteins into the cytosol [8, 19–21]. The mitochondrial NADH dehydrogenase complex (MT-ND) is the main source of reactive oxygen species like H_2_O_2_ in the cell [22]. The release of reactive oxygen species from the mitochondria apparently impacts the capacity for viral replication and may help regulate a pathway towards apoptosis in response to viral infection [23]. Genetic knockout, overexpression and radical scavengers indicate that the MT-ND complex and the production of reactive oxygen species in the mitochondria play a role in governing viral replication [16, 18, 24, 25]. The Tumor Necrosis Factor (TNF) response to viral infection triggers the mitochondrial activity that oxidizes luminol in the presence of reactive oxygen species [26, 27]. Infection by SARS-CoV-2 may lead to the loss of mitochondrial components into the extracellular space [28]. The cytochrome complex members including cytochromes like CYB, CYC, CYTB, cytochrome oxidase (MT-COX) and MT-ND from the mitochondria may play a role in H_2_O_2_ dependent luminol oxidase activity in vivo or in vitro [27, 29–32].

Plasma from COVID-19 patients was compared to ICU patients in Acute Respiratory Distress (ICU-ARDS) and Normal Human plasma EDTA using manual biophysical and biochemical sample preparation. The detection of human plasma proteins by precipitation, preparative quaternary amine chromatography, tryptic digestion and collection by micro C18 disposable resin (ZipTip) for immediate acid dilution and manual injection for nano spray liquid chromatography and tandem mass spectrometry of peptides (LC–ESI–MS/MS) is a laborious but effective means to attain sensitivity in blood plasma analysis [33, 34]. Here, the analysis of protein observation frequency of tryptic peptides by tandem mass spectrometry [35] alongside random and noise MS/MS spectra led to the discovery of new prognostic molecules specific to severe lung damage from COVID-19 infection and ICU-ARDS, such as mitochondrial cytochrome components. The experiments discovered components of the cytochrome electron transport system including CYCB, CYTB, CYC, CYP, MT-ND5 and MT-COX that were increased in the plasma of COVID-19 patients compared to normal controls. PCR analysis of mitochondrial MT-ND1 DNA, LC–ESI–MS/MS of plasma proteins and luminol oxidase assays were consistent to indicate the presence of macromolecule components associated with the cytochrome system in the plasma of COVID-19 patients.

## Materials and methods

### Materials

The Dionex UltiMate 3000 series UHPLC, C18 Acclaim PepMap NanoLC column (75 μm ID, 25 cm length C18), Fusion Lumos Q-Orbitrap-LTQ Tribrid MS (OIT) tandem mass spectrometer [36], LTQ XL linear ion trap (LIT) mass spectrometer [37] and Trizol reagent was from Thermo Fisher Scientific (Waltham, MA, USA). The 1100 HPLC was from Agilent (Santa Clara, CA, USA). Trypsin, salts, buffers, and luminol, H_2_O_2_, 4-iodophenylboronic acid (4IPBA) were obtained from Sigma Aldrich (St. Louis, MO, USA). The quaternary amine (QA) resin on a ceramic support was from BIORAD (Hercules, CA, USA). The HPLC grade water, ethanol, acetone and acetonitrile were obtained from Caledon Laboratories (Georgetown, Ontario, Canada). The 0.45 micron PVDF was from Millipore (Burlington, MA, USA). The imaging workstation was from BIORAD (Hercules, CA, USA).

### Plasma sample collection

The presence of SARS-CoV-2 infection was confirmed by nasal PCR and serology assays at St. Michael’s Hospital, Toronto. EDTA plasma tubes were rapidly inverted 5 times before packing in ice [38]. The ice-cold plasma was then separated from blood cells at ≤ 3,000 RCF for 15 min at 4 °C prior to aliquoting to ≥ 1 ml prior to freezing at − 80 °C prior to analysis. Plasma from COVID-19 patients was compared to acute respiratory distress (ARDS) patients in the ICU and normal healthy volunteers under ethics protocol REB# 20-078. There were 16 COVID positive patients by nasal PCR and serology that were sampled three times, 2 individuals sampled twice and 1 individual sampled once, for a total of 19 individuals. A total of 16 ICU patients in acute respiratory distress were each sampled three times. Normal human plasma (NHP) contained 31 samples including 15 healthy normal volunteers from St. Michael’s hospital and a reference set of 16 normal human plasma from the International Biobank of Luxembourg of the Luxembourg Institute of Health (LIH) collected under a Comité National d’Ethique de Recherche (CNER) Protocol 201107/02 “Biospecimen Research” at the Centre Hospitalier de Luxembourg [39]. Five individuals with COVID and 4 normal plasma were analyzed by high-resolution OIT as an analytical reference. Subsequently, aliquots were thawed on ice, centrifuged at 12,000 RCF for 5 min at 4 °C to separate any cellular debris, and aliquoted to 25 µl samples on ice and refrozen for following analysis. Plasma samples of 25 μl were stored on ice and used for protein analysis.

### Mitochondrial PCR

The MT DNA was extracted from human plasma using the phenol/guanidine isothiocyanate method with the commercial product Trizol. The ND1 forward primer was ND1F 5′-ACTACAACCCTTCGCTGACG-3′ and the reverse primer ND1R was 5′-GAAGAATAGGGCGAAGGGGC-3′ that together yield an expected product of 538 bp [19]. The PCR [40] conditions were: 40 cycles, lid temp 105 °C, 25 µL reaction volume, 94 °C melting (30 s), 58 °C annealing (1 min), 72 °C extension (1 min). The PCR products were separated by a 1.5% Agarose gel run at 100 V for 2 h and stained with GelRed by a minor modification of the method of Huang et al. [41].

### Plasma precipitation

Plasma samples (25 µL) were precipitated with 9 volumes of acetonitrile 90% final (v/v) and centrifuged at 12,000 RCF for 15 min at room temperature. The acetonitrile was removed with a pipette and pelleted material dried under vacuum overnight. The proteins were then resuspended in 250 µL of 20 mM Tris pH 8.85 on ice with occasional vortex, brief centrifugation at 14,000 RCF, and the dissolved proteins were collected from insoluble components with a pipette. The resuspended proteins were assayed for protein content using the Dumbroff assays against BSA standards [42].

### Quaternary amine (ammonium) chromatography

The precipitated, dried and re-dissolved plasma proteins were diluted in 250 µL of 20 mM Tris pH 8.85 and loaded on quaternary amine resin, washed with 5 column volumes of loading buffer and eluted in 200 µL of 300 mM NaCl with 20 mM Tris pH 8.85 [43].

### Tryptic digestion

Tryptic digestion was performed in 600 mM urea and 5% ACN at 1/100 trypsin to protein overnight in 20 mM Tris pH 8.5. The samples were then reduced in 2 mM DTT for 20 min at 50 °C. The samples were digested again at 1/100 trypsin to protein for 2 h and quenched with 5% acetic acid.

### LC–ESI–MS/MS

The plasma from COVID-19, ICU patients and the normal samples were analyzed in technical triplicate. Proteins from human blood fluid that were precipitated in acetonitrile (ACN) [44], re-dissolved 20 mM tris pH 8.85 and collected over preparative quaternary amine ion exchange resin [43] and digested to fully tryptic peptides, collected over preparative C18 ZipTip resin in 5% formic acid and were diluted in 18 µL of 5% formic acid immediately prior to injection via a 20 µL loop [45]. A total of ~ 5 µg of extracted and purified peptides was injected for each analytical HPLC separation over a 150 micron ID column (15 cm) with inline filter frits. The peptides were ionized by nano spray of the solvent gradient generated at 2 µL per minute split to a flow of ~ 200 nL per minute with a transfer capillary temperature of ≤ 250 °C into a Thermo Electron Corporation LTQ XL linear ion trap (LIT) mass spectrometer [37]. The peptides were randomly and independently sampled from 150 to 2000 m/z as the peptides eluted from the HPLC column into the nano electrospray source. A reference database was created using high-resolution trihybrid mass spectrometry where identical COVID-19 and NHP samples were analyzed by UPLC using Dionex UlitMate 3000 series over an Acclaim PepMap 100 C18 HPLC column (Thermo) C18, 2uM, ID: 0.075 mm × 250 mm for the orbital ion trap (OIT) [36, 46].

### Peptide MS/MS spectra correlation analysis

The LC–ESI–MS/MS spectra and the results of the correlation algorithms were parsed into an SQL Server Database for analysis with the R system [33]. A physical filter of at least one thousand (E3) intensity counts for peptide parent ions was used to limit noise MS/MS spectra. The MS/MS spectra were fit to the peptides of the non-redundant human UNIPARC human proteins. The MS/MS spectra were fit by fully tryptic enzyme specification with a charge state of 2^+^or 3^+^ with up to three missed cleavages by SEQUEST [47], and X!TANDEM [48] using the default ion trap setting of fragments within 0.5 Da and within ± 3 m/z for the calculated peptide [M + H]^+^ [33, 34, 49–52]. The MS/MS spectra were fit to fully tryptic or phosphotryptic peptides by SEQUEST and to optional phospho/tryptic peptides by X!TANDEM. All the Gene Symbols presented showed p-values and FDR corrected q-values of < 0.01 from the Chi Square comparison of authentic observation frequency to that of the Monte Carlo simulation with computer generated random MS/MS spectra and random MS/MS from blank injection noise [34, 39, 50–54].

### Computational analysis in SQL and statistical analysis with R

The LC–ESI–MS/MS results from samples and blank controls together with the results of the X!TANDEM and SEQUEST algorithms were collected and redundancy filtered out in SQL Server followed by statistical analysis performed with the R statistical system and biological connections represented using STRING algorithm [55]. The total number of MS/MS spectra from precursors greater than E3 intensity was used to normalize the observation frequency from the SEQUEST algorithm between the COVID-19, ICU-ARDS and NHP treatments for summation and Chi Square χ^2^ comparisons. The number of manually prepared samples and manual injection LC–ESI–MS/MS runs for each treatment were: Covid positive patients (COVID-19), 165 LC–ESI–MS/MS runs with a sum of 3,116,582 MS/MS spectra ≥ E3 intensity; Normal human plasma (NHP), 93 LC–ESI–MS/MS runs with a sum of 1,846,168 MS/MS spectra ≥ E3 intensity; and ICU acute respiratory distress syndrome (ARDS) control, 144 LC–ESI–MS/MS runs with a sum of 2,746,085 MS/MS spectra ≥ E3 intensity. The observation frequency for the ICU-ARDS and NHP treatments from the random and independent sampling of all plasma in triplicate by the LIT was corrected by the equations:

1 $$ {\text{ICU Observation frequency}} = {\text{ICU Count}}*\left( {\Sigma {\text{COVID MS/MS/}}\Sigma {\text{ICU MS/MS}}} \right) $$

2 $$ {\text{NHP Observation frequency}} = {\text{NHP count}}*\left( {\Sigma {\text{COVID MS/MS / }}\Sigma {\text{NHP MS/MS}}} \right) $$

### Luminol oxidase activity

Cytochrome such as CYB, CYC, CYTB, CYP and the electron transport components such as the MT-COX and MT-ND proteins observed in the plasma have been previously shown to play a role in the reaction of luminol in the presence of H_2_O_2_ to yield a chemiluminescent signal [27, 29–32]. One µL sample of the plasma proteins, alongside known amounts of Cytochrome C preparation in the same buffer, was spotted directly onto PVDF using a pipette [56]. The total luminol oxidase enzyme activity was measured using ECL solution containing 4-iodophenylboronic acid (4IPBA) (100 mM Tris/HCl pH 8.85, 2.5 mM luminol, 0.4 mM 4IPBA, 2.6 mM Hydrogen peroxide) that generated strong specific signals and low background [57] on a BIORAD image analysis workstation.

## Results

### Mitochondrial PCR

**Fig. 1:**
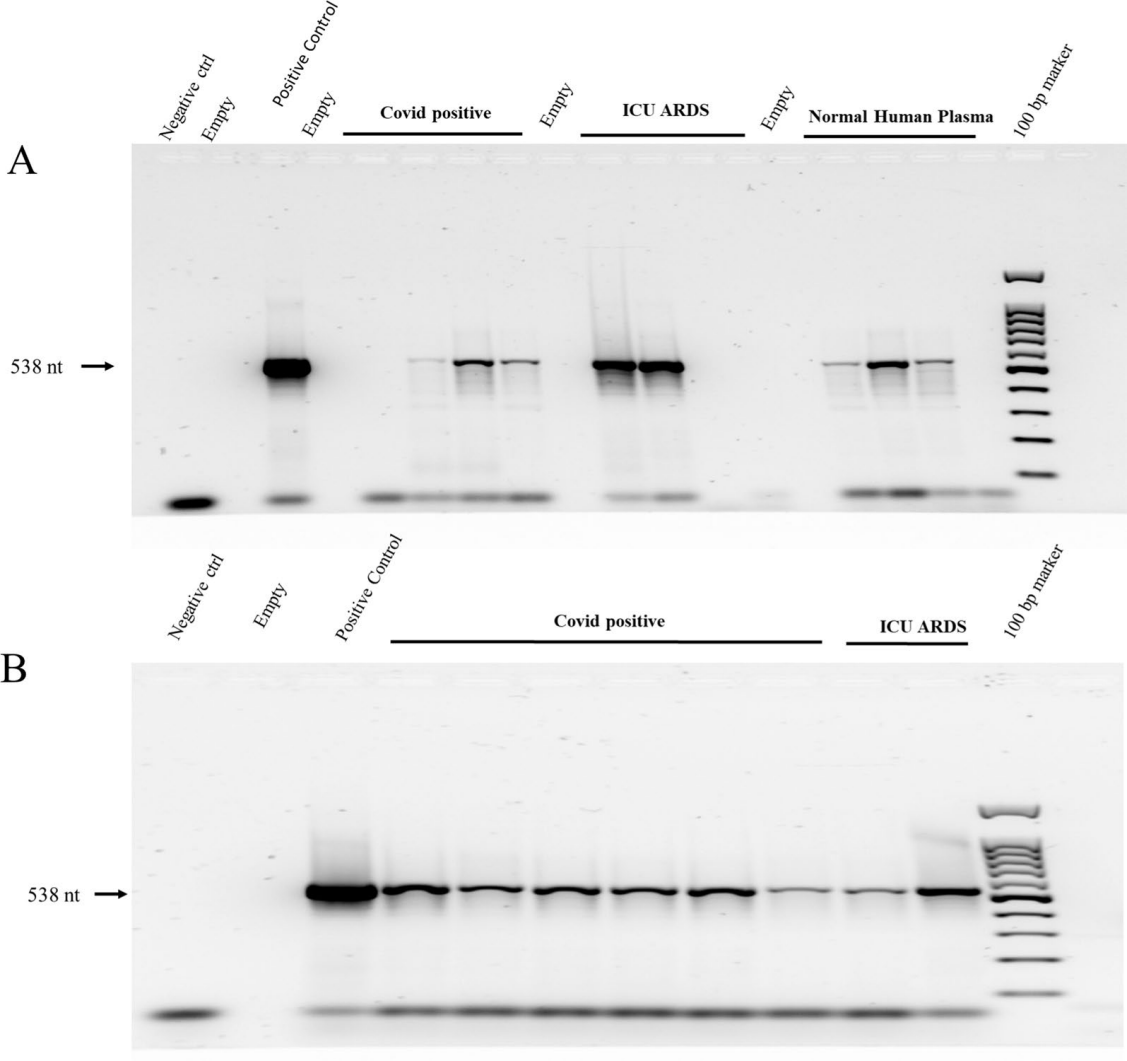
Agarose gel electrophoresis and GelRed Staining of PCR products from mitochondrial DNA in human plasma with mitochondrial MT-ND1 specific primers. **A** PCR analysis of representative COVID-19, ICU-ARDS and NHP plasma samples; **B** the variation in COVID-19 and ICU-ARDS PCR amplification. The PCR conditions were 40 cycles, lid temp 105 °C, 25 µL reaction volume, 94 °C melting (30 s), 58 °C annealing (1 min), 72 °C extension (1 min). Template DNA was extracted from plasma with Trizol. Serum from the Luxembourg cohort was used as Normal Human Plasma (NHP) healthy controls

The polymerase chain reaction (PCR) [40] showed mitochondrial DNA encoding the cytochrome electron transport complex component NADH dehydrogenase 1 (MT-ND1) was detectable in the plasma of most COVID-19 as well as ICU-ARDS patients. The presence of MT-ND1 DNA in the plasma was also detected in some normal human plasma (Fig. 1A). MT-ND1 DNA showed variation across individual COVID-19 and ICU-ARDS patients after PCR amplification, agarose electrophoresis and fluorescent staining (Fig. 1B) in agreement with previous results [19]. Thus macromolecule components from the mitochondria were clearly observed in the plasma of COVID-19 and ICU-ARDS patients as well as NHP controls.

### Comparison of COVID-19 vs NHP and ICU-ARDS

The observation frequency of proteins from COVID-19 plasma was compared to those of ICU-ARDS and/or Normal Human Plasma (NHP) by One Way ANOVA, and the Chi Square test χ2, which revealed some proteins showed significant variation in the plasma across the disease and control treatments. All proteins reported herein were shown to have a low rate of type I false positive identification of p ≤ 0.01 versus the Monte Carlo simulation of random MS/MS from computer random numbers or random physical noise with FDR values q ≤ 0.01 [50, 52–54]. Analysis of COVID and normal samples by nano electrospray with a tri-hybrid orbital trap, a highly resolving mass spectrometer, showed good agreement on many of the proteins identified with the linear ion trap (LIT).

### Plasma proteins

The majority of human protein gene symbols from serum proteins showed nearly identical observation frequency between COVID-19 versus normal controls (NHP) or severe respiratory distress ICU-ARDS. Plasma proteins including AAT, ABO, APP, FGA, HPX, ITIH4, PON and others showed similar or higher observation frequency in NHP compared to COVID-19 (Table 1).

**Table 1:** A comparison of corrected mean observation frequency per gene symbol for common serum proteins and acute phase response proteins in EDTA plasma across COVID-19, ICU-ARDS and Normal Human Plasma (NHP)

Gene_symbol	COVID-19				COVID-19				COVID-19				ICU-ARDS			
	X!TANDEM				SEQUEST				SEQUEST				X!TANDEM			
	TRYP/STYP	Delta	χ2		TRYP	Delta	χ2		STYP	Delta	χ2		TRYP/STYP	Delta	χ2	
AANAT	2	-3	2		8	5	6		48	11	3		1	-4	3	
AAT	48	-77	47		155	-228	135		13	5	3		148	23	4	
ABO	0	0	0		22	7	3		33	10	4		0	0	0	
ACHE	5	3	3		65	18	7		105	48	40*		5	3	3	
ALB	6022	398	28*		105,303	14,524	2324*		19,572	2942	520*		5843	219	9*	
APCS	311	279	2359*		640	472	1318*		134	65	60*		174	142	611*	
APP	2	0	0		26	11	8		39	11	4		1	-1	0	
AZGP1	451	363	1481*		944	742	2712*		89	57	98 *		153	65	47*	
B2M	57	56	1568*		268	233	1508*		10	0	0		48	47	1105*	
CRP	151	151	22,801*		618	611	46,665*		180	161	1296*		234	234	54,756*	
FGA	236	-522	359		636	-1141	732		410	-569	330		528	-230	70	
HBA1	128	64	63*		336	192	254*		72	54	153*		149	85	111*	
HP	896	234	83*		5613	2910	3132*		752	403	464*		1249	587	520*	
HPR	574	183	85*		3203	1841	2487*		1302	757	1050*		788	397	402*	
HPX	217	-235	122		863	-1186	686		226	-217	106		538	86	16*	
HuSAA1g	0	0	0		3	1	0		11	9	27*		0	0	0	
ITIH4	24	-119	98		190	-333	212		134	-70	24		75	-68	32	
KLKB1	0	0	0		59	27	22*						0	0	0	
LU	0	0	0		90	40	31*		95	21	6		0	0	0	
ORM1	1642	1363	6635*		12,752	11,529	108,593*		2525	2294	22,683*		1278	999	3564*	
ORM2	894	732	3287*		9138	8350	88,368*		2763	2479	21,563*		630	468	1344*	
PON1	1	-7	5		14	-32	22		14	-8	3		2	-6	4	
PON2	3	3	9		35	-3	0		50	-7	1		2	2	4	
PON3	1	1	1		22	-3	0		16	-9	3		5	5	25*	
RBP4	270	177	333*		1570	1093	2499*		331	147	117 *		183	90	86*	
RhCE	0	0	0		16	11	20*		17	14	49*		0	0	0	
RHD	0	0	0		26	-16	6		7	2	1		0	0	0	
RhDTI	0	0	0		37	-27	11		0	0	0		0	0	0	
RhIVb(J)	0	0	0		32	-30	14		0	0	0		0	0	0	
S100A10	9	4	3		97	26	9*		149	-3	0		5	0	0	
S100A14	0	-2	1		85	55	98*		34	-3	0		0	-2	1	
S100A7A	1	-2	1		46	22	19*		50	13	4		0	-3	2	
S100A8	5	3	3		95	53	65*		29	14	12*		37	35	408*	
S100A9	32	30	300*		78	76	1925*		18	11	15*		91	89	2640*	
SAA1	326	312	6490*		895	869	27,969*		0	0	0		545	531	18,797*	
SAA2	150	141	1988*		525	508	14,337*		0	0	0		290	281	7896*	
SAA2-SAA4	136	-16	2		506	306	466*		0	0	0		284	132	114*	
SAA4	3	-139	135		23	-166	145		0	0	0		11	-131	120	
SAAL1	0	0	0		33	-13	4		23	-9	2		0	0	0	
TF	323	-494	298		1116	-2446	1679		180	-124	50		860	43	2	
TFRC	1	-2	1		13	1	0		34	14	9		2	-1	0	

Gene_symbol	ICU-ARDS				ICU-ARDS				NHP			OIT		
	SEQUEST				SEQUEST				X!TANDEM	SEQUEST		X!TANDEM	SEQUEST	
	TRYP	Delta	χ2		STYP	Delta	χ2		TRYP/STYP	TRYP	STYP	TRYP/STYP	TRYP	STYP
AANAT	8	5	6		27	-10	3		5	3	37	0	2	12
AAT	510	127	42*		24	16	28*		125	383	8	41	105	4
ABO	25	10	6		30	7	2		0	15	23	0	2	15
ACHE	41	-6	1		59	2	0		2	47	57	2	5	5
ALB	113,793	23,014	5834*		21,879	5249	1657*		5624	90,779	16,630	1307	11,487	2291
APCS	387	219	284*		54	-15	3		32	168	69	137	236	15
APP	16	1	0		23	-5	1		2	15	28	0	12	11
AZGP1	340	138	94*		47	15	7		88	202	32	175	348	25
B2M	190	155	667*		12	2	0		1	35	10	9	23	1
CRP	864	857	91,806*		300	281	3948*		0	7	19	19	39	15
FGA	1574	-203	23*		1133	154	24*		758	1777	979	255	382	102
HBA1	384	240	397*		127	109	625*		64	144	18	54	103	42
HP	8743	6040	13,492*		1089	740	1565*		662	2703	349	262	794	105
HPR	4700	3338	8175*		1833	1288	3038*		391	1362	545	99	528	209
HPX	2275	226	25*		508	65	10*		452	2049	443	181	629	68
HuSAA1g	5	3	3		9	7	16*		0	2	2	0	0	3
ITIH4	281	-242	112		161	-43	9		143	523	204	247	433	26
KLKB1	52	20	12*						0	32		0	39	0
LU	98	48	45*		126	52	36*		0	50	74	0	13	1
ORM1	10,140	8917	64,962*		2298	2067	18,416*		279	1223	231	355	1589	173
ORM2	7269	6481	53,236*		2500	2216	17,230*		162	788	284	261	1019	264
PON1	15	-31	20		8	-14	9		8	46	22	28	35	16
PON2	20	-18	8		47	-10	2		0	38	57	0	6	5
PON3	17	-8	2		18	-7	2		0	25	25	0	8	1
RBP4	772	295	182*		213	29	5		93	477	184	86	564	100
RhCE	16	11	20*		9	6	9		0	5	3	0	0	2
RHD	30	-12	3		6	1	0		0	42	5	0	1	2
RhDTI	44	-20	6		0	0	0		0	64	0	0	1	0
RhIVb(J)	31	-31	15		0	0	0		0	62	0	0	1	0
S100A10	62	-9	1		93	-59	23		5	71	152	0	20	12
S100A14	49	19	12*		24	-13	4		2	30	37	0	0	1
S100A7A	51	27	29*		35	-2	0		3	24	37	0	18	39
S100A8	226	184	787*		81	66	272*		2	42	15	9	38	10
S100A9	246	244	19,845*		36	29	105*		2	2	7	25	62	16
SAA1	1451	1425	75,208*		0	0	0		14	26	0	77	99	0
SAA2	880	863	41,376*		0	0	0		9	17	0	28	39	0
SAA2-SAA4	876	676	2274*		0	0	0		152	200	0	51	66	0
SAA4	40	-149	117		0	0	0		142	189	0	31	41	0
SAAL1	33	-13	4		24	-8	2		0	46	32	0	5	1
TF	2968	-594	99		431	127	53		817	3562	304	246	1053	126
TFRC	17	5	2		38	18	15		3	12	20	0	3	3

The SQL SERVER accession descriptions that contained “sera” or “serum” or “sero” or “plasma” not “membrane” were queried. The observation frequency was computed after correcting for the number of MS/MS spectra in each treatment with precursor intensity ≥ E3 detector counts. The spectra were fit to tryptic peptides (TRYP) or optionally phospho/tryptic (STYP) peptides by the X!TANDEM algorithm. The peptides were fit to tryptic peptides (TRYP) or obligate phosphotryptic peptides by the SEQUEST algorithm. The difference (Delta) and Chi Square value χ2 with respect to NHP are shown. The asterisk * indicates a significant increase in observation frequency by the Chi Square test in the COVID-19 or ICU-ARDS plasma compared to the Normal Human Plasma (NHP) with χ2 ≥ 9, p ≤ 0.01

### Acute phase plasma proteins

A small minority of proteins that included acute phase markers increased in COVID-19 versus the controls based on counts of tryptic (TRYP) or phospho/tryptic (STYP) peptides with Chi-Square score of greater than 800 (χ^2^ ≥ 9, p ≤ 0.01) (Table 1). The observation frequency of the acute response serum proteins such as AZGP1, B2M, CRP, HP, HPR, ORM, RBP4, and some SAA was dramatically increased in COVID-19 compared to NHP plasma and showed agreement with previous studies [1–11]. In contrast AAT and transferrin (TF) showed a sharp decline in COVID-19. However, the observation frequency of CRP, AAT, FGA, S100, SAA1 and others was often greater in ICU-ARDs or COVID-19 compared to normals, therefore these proteins were not specific markers of COVID-19 infection but rather reflected lung damage (Table 1).

### Apolipoproteins

Many apolipoproteins were dramatically reduced in COVID-19 compared to NHP or ICU-ARDS plasma. The apolipoproteins APOA1, APOA2 and APOA4 showed a 30% to 90% decline in COVID-19 and/or ICU-ARDS observation frequency compared to Normals (NHP). The deficiency in apolipoproteins levels in COVID-19 compared to normal human plasma (NHP) and ICU-ARDS patients was most pronounced for APOA1, APOA2, APOA4, APOC3 and APOE (Table 2). In contrast, APOBR was apparently increased in ICU-ARDS. Some proteins such as APOA1BP binding protein were constant over all treatments.

**Table 2:** A comparison of corrected mean observation frequency per gene symbol of apolipoproteins across COVID-19, ICU-ARDS and Normal Human Plasma (NHP)

Gene_Symbol	COVID-19	COVID-19	COVID-19	ICU-ARDS	ICU-ARDS	ICU-ARDS	NHP	NHP	NHP	OIT	OIT	OIT
	X!TANDEM	SEQUEST	SEQUEST	X!TANDEM	SEQUEST	SEQUEST	X!TANDEM	SEQUEST	SEQUEST	X!TANDEM	SEQUEST	SEQUEST
	TRYP/STYP(%NHP)	TRYP(%NHP)	STYP(%NHP)	TRYP/STYP(%NHP)	TRYP(%NHP)	STYP(%NHP)	TRYP/STYP	TRYP	STYP	TRYP/STYP	TRYP	STYP
APOA	0(0)	24(75)	3(100)	1(-)	53(166)	3(100)	0	32	3	0	2	0
ApoA-IV	0(0)	1(-)	1(-)	0(0)	0(0)	1(-)	0	0	0	0	0	0
APOA1	476(18)	1061(14)	176(11)	1862(72)	5519(74)	1162(75)	2588	7440	1546	374	652	86
APOA1BP	3(100)	35(113)	52(95)	1(33)	33(106)	48(87)	3	31	55	3	9	7
APOA2	76(27)	284(26)	12(150)	218(76)	825(75)	16(200)	285	1103	8	60	116	6
APOA4	38(7)	134(12)	89(23)	346(68)	776(70)	291(75)	510	1111	390	272	550	142
APOA5	1(-)	22(88)	45(141)	2(-)	22(88)	32(100)	0	25	32	3	3	6
APOB	3(20)	146(97)	168(114)	5(33)	154(103)	186(126)	15	150	148	24	52	27
apoB28.1	0(0)	1(50)	6(60)	0(0)	2(100)	5(50)	0	2	10	0	1	5
APOB48R	10(-)	206(94)	473(108)	9(-)	293(134)	598(137)	0	219	437	2	49	72
APOBEC1	3(75)	18(225)	13(118)	2(50)	11(138)	7(64)	4	8	11	0	5	8
APOBEC2	0(0)	29(242)	22(76)	2(100)	24(200)	25(86)	2	12	29	0	2	3
APOBEC3A	0(0)	13(54)	9(90)	2(67)	18(75)	15(150)	3	24	10	0	1	3
APOBEC3A_B	0(0)	13(65)	8(80)	2(100)	18(90)	15(150)	2	20	10	0	4	3
APOBEC3B	1(-)	24(65)	22(88)	1(-)	20(54)	25(100)	0	37	25	0	1	4
APOBEC3C	0(0)	10(71)	11(110)	0(0)	10(71)	3(30)	0	14	10	0	4	1
APOBEC3D	1(-)	38(88)	61(117)	0(0)	34(79)	59(113)	0	43	52	1	6	5
APOBEC3F	0(0)	22(65)	25(114)	0(0)	21(62)	16(73)	0	34	22	0	0	0
APOBEC3G	1(-)	88(55)	40(143)	1(-)	63(40)	42(150)	0	159	28	0	1	0
APOBEC3H	0(0)	20(83)	24(171)	0(0)	16(67)	15(107)	2	24	14	0	3	4
APOBEC4	1(-)	19(136)	33(138)	1(-)	23(164)	45(188)	0	14	24	0	1	1
APOBR	11(-)	221(105)	484(109)	10(-)	297(141)	619(139)	0	211	446	2	50	72
APOC1	0(0)	9(11)	9(113)	7(27)	59(75)	5(63)	26	79	8	25	23	2
APOC2	6(27)	13(37)	5(500)	34(155)	52(149)	1(100)	22	35	1	16	8	0
APOC3	20(12)	62(21)	8(160)	95(58)	206(70)	5(100)	165	296	5	31	41	1
APOC4	21(175)	21(210)	21(700)	22(183)	16(160)	10(333)	12	10	3	1	3	1
APOC4-APOC2	30(81)	37(61)	18(360)	65(176)	100(164)	5(100)	37	61	5	22	16	1
APOD	5(12)	37(32)	21(51)	38(90)	105(90)	34(83)	42	117	41	42	86	12
APOE	1(2)	39(21)	49(39)	6(11)	60(33)	76(60)	56	184	127	35	79	35
APOF	1(50)	15(75)	28(350)	2(100)	14(70)	11(138)	2	20	8	8	18	5
APOH	6(38)	86(25)	59(42)	16(100)	212(62)	64(46)	16	344	140	17	216	97
APOL1	4(24)	56(98)	52(91)	5(29)	41(72)	43(75)	17	57	57	14	33	16
APOL2	0(0)	35(109)	63(115)	0(0)	35(109)	45(82)	0	32	55	1	5	14
APOL3	1(100)	25(156)	45(180)	3(300)	11(69)	16(64)	1	16	25	1	2	2
APOL4	0(0)	20(71)	28(112)	2(200)	15(54)	26(104)	1	28	25	0	7	9
APOL5	2(67)	78(94)	84(83)	3(100)	63(76)	68(67)	3	83	101	0	12	23
APOL6	0(0)	78(91)	130(118)	0(0)	77(90)	117(106)	3	86	110	0	4	5
APOLD1	0(0)	18(86)	48(112)	0(0)	27(129)	52(121)	2	21	43	1	7	5
APOM	9(43)	22(33)	27(100)	25(119)	20(30)	21(78)	21	66	27	6	17	0
APOO	0(0)	17(63)	28(311)	2(-)	19(70)	27(300)	0	27	9	0	1	2
APOOL	3(150)	62(111)	197(158)	2(100)	75(134)	226(181)	2	56	125	0	21	4
APOPT1	1(-)	37(116)	44(142)	1(-)	35(109)	30(97)	0	32	31	1	2	5

The observation frequency was computed after correcting for the number of MS/MS spectra in each treatment with precursor intensity ≥ E3 detector counts. The SQL SERVER gene symbols were queried by “Apolipo”. The percent value with respect to NHP (%NHP) are shown

### Mitochondrial and cytochrome proteins

Mitochondrial and cytochrome proteins were observed to be elevated in the plasma of COVID-19 patients versus the ICU-ARDS and NHP controls. The observation frequency of ICU-ARDS and NHP treatments was computed after correcting for the number of MS/MS spectra in each treatment with precursor intensity ≥ E3 detector counts to ensure a balanced comparison [58]. Mitochondrial proteins such as ATP5A1, CYB561D1, several CYP accessions, L2HGDH, two MRP, MRPL37, NDUFS1 and others showed increased observation frequency across COVID-19 and ICU-ARDS versus NHP individually by the Chi Square test (χ2 ≥ 10, p ≤ 0.01) and as a group by one way ANOVA (p ≤ 0.003) (Table 3). Chi Square analysis of corrected observation frequency indicated that cytochrome complex members including CYB, CYTB, CYP, CYBR, MT-COX, NDUF, MT-ND5 and other structurally or functionally related proteins were significantly elevated (χ^2^ ≥ 21, DF = 1, p ≤ 0.0001) in COVID-19 plasma (Table 4). The observation frequency of mitochondrial or cytochrome proteins from BFPS peptides typically showed a two fold to three fold increase that was significant by the Chi Square test (p ≤ 0.01). For example, MRPL37 showed an increased observation frequency from 216 tryptic peptides from SEQUEST in the NHP samples to 760 observations in COVID-19 that is an increase of more than threefold with a χ^2^ value of χ^2^ = 1364 where χ^2^ = 9 is the cut off for significance. The cytochrome CYP3A43 was observed in NHP 75 times from BFPS tryptic peptides by the SEQUEST algorithm but was observed 192 times in COVID-19 plasma that is a greater than two-fold increase with a highly significant Chi Square value (χ^2^ = 180) where a χ^2^ of ≥ 9 is significant.

**Table 3:** A comparison of corrected mean observation frequency of tryptic (TRYP) versus phosphotryptic (STYP) mitochondrial and cytochrome proteins per gene symbol from SEQUEST across COVID-19, ICU-ARDS and Normal Human Plasma (NHP)

Gene_Symbol	COVID-19			COVID-19			ICU-ARDS			ICU-ARDS			NHP	NHP	OIT	
	TRYP	Delta	χ2	STYP	Delta	χ2	TRYP	Delta	χ2	STYP	Delta	χ2	TRYP	STYP	TRYP	STYP
MRPL37	760	544	1364*	238	80	40*	398	182	153*	142	-16	2	216	158	6	23
L2HGDH	672	261	165*	932	247	89*	562	151	55*	864	179	47*	411	685	5	4
ACSS3	471	77	15*				547	153	59*				394		36	
CYP3A7-CYP3A51P	223	93	66*				210	80	49*				130		6	
ATP5A1	219	65	27*	116	32	12*	182	28	5	121	37	16*	154	84	21	5
AASS	206	44	12*	417	84	21*	211	49	15*	539	206	127*	162	333	21	22
POLRMT	201	46	14*				196	41	11*				155		31	
MRPS16	200	143	353*	277	146	161*	157	100	172*	292	161	196*	57	131	2	18
MRPS27	196	59	25*	93	51	60*	211	74	40*	64	22	11*	137	42	6	13
CYP3A4	192	117	180*	77	30	19*	158	83	91*	97	50	52*	75	47	8	10
CYP3A43/CYP3A4	187	116	187*	106	37	20*	154	83	96*	135	66	62*	71	69	8	15
ACAD9	186	95	98*	253	116	98*	179	88	84*	169	32	7	91	137	25	9
GUF1	177	61	32*				157	41	14*				116		22	
CYP3A43	175	91	97*	65	23	12*	143	59	41*	56	14	5	84	42	8	9
ACSF3	173	45	16*				117	-11	1				128		18	
GPAT2	169	103	158*				63	-3	0				66		9	
CYP17A1	166	43	15*				134	11	1				123		10	
COA6	163	78	71*				130	45	24*				85		8	
NDUFS1	163	39	12*	67	20	8	133	9	1	52	5	1	124	47	19	10
ACSM5	158	62	40*				133	37	14*				96		13	
ND5	157	149	2467*				118	110	1344*				8		0	
BDH1	155	50	24*	242	115	103*	123	18	3	183	56	25*	105	127	6	7
CYB561D1	152	108	259*	13	8	11*	203	159	562*	9	4	3	44	5	0	0
MCCC1	152	52	27*				165	65	42*				100		20	
GPD2	150	34	10*				125	9	1				116		10	
DHODH	148	39	14*	118	57	52*	94	-15	2	35	-26	11*	109	61	14	1
IMMT	124	52	37*				153	81	90*				72		58	
LOC101060817	122	85	190*				81	44	51*				37		1	
TIMM21	119	33	13*				109	23	6				86		7	
MIPEP	115	29	10*				81	-5	0				86		17	
ACAD8	114	35	15*	194	90	77*	111	32	13*	176	72	49*	79	104	4	10
DLD	114	32	12*				108	26	8				82		15	
CYP1A1	107	39	22*				76	8	1				68		4	
CYP21A2	107	50	43*	18	13	28*	89	32	18*	6	1	0	57	5	27	0
CYP39A1	107	55	57*				93	41	32*				52		3	
GCSH	103	79	250*	47	32	64*	62	38	58*	24	9	5	24	15	1	10
CYP26B1	99	40	27*				67	8	1				59		11	

The observation frequency was computed after correcting for the number of MS/MS spectra in each treatment with precursor intensity ≥ E3 detector counts. The SQL SERVER accession descriptions were queried by “mitochondria” or “cytochrome”. The difference (Delta) and Chi Square value χ2 with respect to NHP are shown. The asterisk * indicates a significant increase in observation frequency by the Chi Square test in the COVID-19 or ICU-ARDS plasma compared to the Normal Human Plasma (NHP) with χ2 ≥ 10, p ≤ 0.01. The chance that the observation frequency of the mitochondrial and cytochrome gene symbols shown are similar between COVID-19, ICU-ARDS and NHP by One Way ANOVA was p ≤ 0.003

**Table 4:** Mitochondrial proteins identified from SEQUEST was compared by Chi-Square between COVID vs ICU and COVID vs NHP from the non-redundant sum of tryptic (TRYP) and optional phosphotryptic (STYP) peptides

Gene Symbol	COVID vs ICU Delta	COVID vs ICU χ2	Gene Symbol	COVID vs NHP Delta	COVID vs NHP χ2	Gene Symbol	ICU vs COVID Delta	ICU vs COVID χ2	Gene Symbol	ICU vs NHP Delta	ICU vs NHP χ2
MRPL58	931	643*	MRPL37	389	922*	CHCHD6	269	333*	NDUFC1	81	418*
MRPL37	273	240*	CARS2	226	341*	SLC25A13	906	299*	CYB561D1	106	379*
MRPL4	153	164*	COX7B2	49	313*	LIPT1	149	147*	PTCD3	67	207*
ALDH1L2	175	105*	MRPS16	145	256*	NDUFC1	62	108*	SLC25A13	725	197*
NUDT9	121	103*	GCSH	60	252*	SLIRP	97	62*	CARS2	149	175*
ALDH2	63	100*	CYB561D1	84	206*	SARDH	53	56*	MRPS16	115	162*
MRPL40	49	99*	IDH2	38	195*	ABCB8	17	30*	MRPS18A	166	160*
MIEF1	58	92*	LOC101060817	85	189*	PTCD3	21	24*	HTRA2	74	158*
DHODH	60	89*	MRPL58	563	186*	NDUFS8	14	21*	RSAD1	86	137*
COX6B2	24	89*	HTRA2	88	180*				MTG2	73	106*
DHTKD1	68	88*	RSAD1	100	171*				COQ8A	162	102*
GATB	51	69*	NLN	107	155*				COX7B2	26	98*
cytb	49	69*	CHCHD10	37	145*				MRPL37	102	90*
ATPAF1	56	68*	HSPA9	76	137*				HSCB	72	89*
NDUFV1	118	67*	SQOR	44	116*				TCAIM	9	81*
SIRT3	62	67*	CYP3A4	66	104*				DARS2	65	80*
SLC25A42	89	65*	CYP3A43/CYP3A4	71	102*				PCCB	65	78*
MALSU1	131	64*	DHTKD1	70	99*				TOMM7	55	76*
TRNT1	79	62*	ACAD9	110	99*				PRDX5	57	75*
XRCC6BP1	28	62*	ALDH2	60	89*				NDUFAF1	91	74*
LOC102724023	105	61*	L2HGDH	173	88*				HSPA9	43	72*
CYTB	18	60*	CYP3A43	72	81*				ATP5O	18	71*
44621	27	58*	GATB	52	76*				ACSS3	156	71*
CYP3A5	33	49*	COA6	78	73*				CYP3A4	57	67*
CYP2C9	47	48*	CYBRD1	28	71*				MTFMT	48	67*
AUH	60	47*	MRPL19	48	70*				CYP3A43/CYP3A4	61	64*
CYP2E1	26	44*	COX6B2	23	70*				AASS	113	63*
COQ2	33	43*	TRAP1	29	67*				IDH2	23	61*
TARS2	45	43*	C14orf159	26	66*				IMMT	73	60*
MRPL32	31	41*	DARS2	62	66*				CHCHD6	139	57*
TFAM	68	40*	SLC25A30	8	64*				GCSH	23	54*
MRPL57	35	40*	TCAIM	8	64*				CHCHD10	20	54*
TDH	20	39*	CYP27B1	44	64*				SLIRP	105	53*
ATP5B	40	37*	CYB5B	28	61*				MRPL49	19	52*
ATP5H	15	37*	ETFA	14	60*				ERAL1	10	52*
COI	6	36*	MRPL52	14	58*				METTL17	33	51*
ATP23	36	35*	BDH1	71	57*				SQOR	34	49*
TOMM5	13	35*	MRPS18A	105	56*				CLPX	34	47*
CHDH	57	34*	TOMM7	33	53*				MRPS22	11	44*
ACAD9	64	34*	GLRX5	44	52*				LOC101060817	38	43*
NDUFV3	38	33*	MRPL1	67	50*				NLN	53	43*
GOT2	46	33*	COQ9	77	49*				SLC25A21	38	43*
PMPCB	57	33*	MRPS25	7	49*				TOP1MT	48	40*
ME3	20	32*	SLC25A21	38	47*				NDUFS8	17	40*
GFM1	53	32*	C2orf47	55	46*				TIMM50	18	38*
SDHB	39	32*	BCS1L	15	46*				COX5B	38	38*
PDF	21	31*	MTFMT	41	43*				CYP2W1	40	37*
RHOT1	18	31*	RHOT2	23	42*				MCCC1	60	36*
CYP11B1	12	31*	AUH	57	42*				PGAM5	19	36*
HIBCH	27	29*	LOC102724023	89	42*				TRAP1	20	35*

The observation frequency was computed after correcting for the number of MS/MS spectra in each treatment with precursor intensity ≥ E3 detector counts. The difference (Delta) and Chi Square value χ2 with respect to NHP are shown. The Chi Square values of observation frequency computed from fully tryptic or phosphotryptic peptides between COVID-19 plasma and ICU-ARDS versus NHP are shown sorted for each comparison. The SQL SERVER accession descriptions were queried by “*mitochondria*” or “*cytochrome*”. The asterisk * indicates a significant increase in observation frequency by the Chi Square test in the COVID-19 or ICU-ARDS plasma compared to the Normal Human Plasma (NHP) with χ2 ≥ 21, p ≤ 0.001

### Mitochondrial protein interactions

**Fig. 2:**
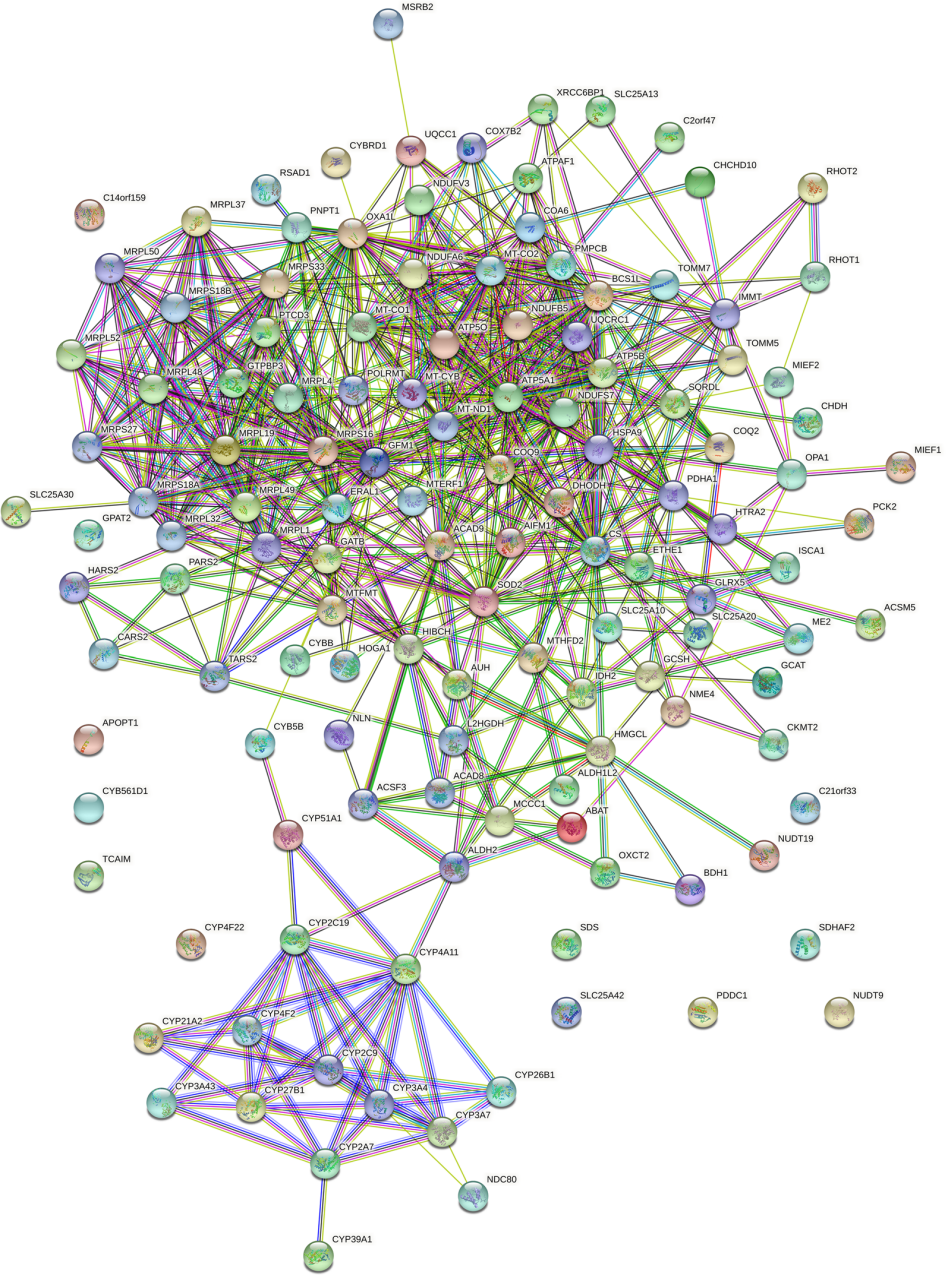
The mitochondrial and cytochrome proteins specific to COVID-19 with a Chi Square value greater than 21 compared to ICU Acute Respiratory Distress Syndrome (ARDS) and/or Normal Human Plasma. The results reported in Table 4 with COVID-19 versus NHP and/or ICU-ARDS of χ^2^ ≥ 21 (p ≤ 0.001) were automatically analyzed by the STRING algorithm. Network Statistics: number of nodes, 131; number of edges, 652; average node degree, 9.95; avg. local clustering coefficient, 0.51; expected number of edges, 86; PPI enrichment p-value, < 1.0e-16

The mitochondria may contain more than 1100 proteins [59]. However only a small subset of 131 mitochondrial proteins was observed to be sharply increased in the plasma of COVID-19 patients. STRING analysis indicated that there were many known functional or structural interactions between the mitochondrial molecules observed (Fig. 2). There were 652 previously established interactions between these 131 mitochondrial proteins indicating that these mitochondrial proteins may form structural or functional protein complexes [60].

### Venn diagram of Chi Square analysis

**Fig. 3:**
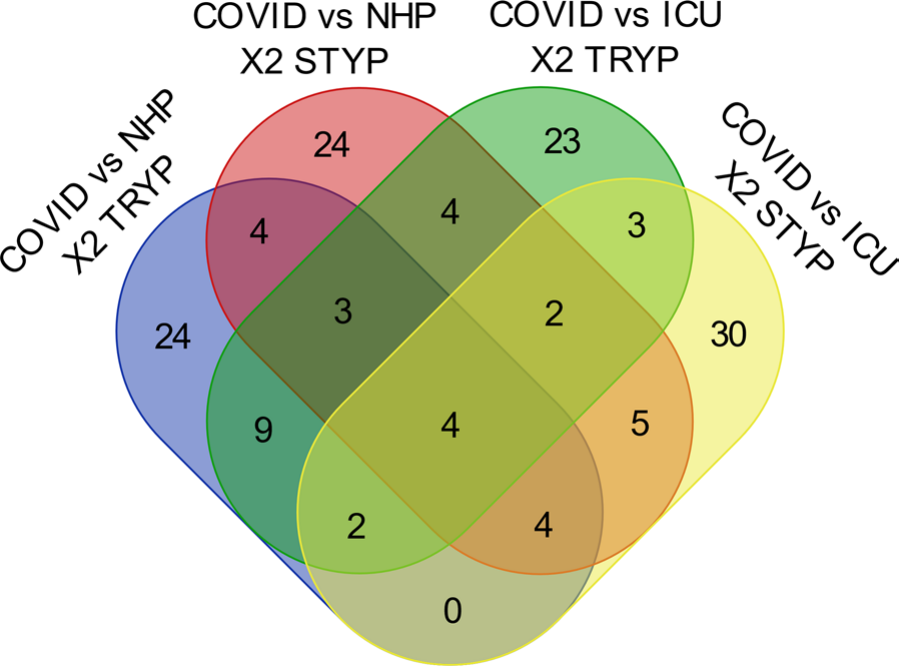
The Venn diagram of the top Chi-Square (χ2 > 21, p ≤ 0.0001) results from COVID-19 versus ICU-ARDS and COVID-19 versus NHP at the level of full tryptic or optionally phospho/tryptic peptides shown in Table 4

A comparison of the proteins in COVID versus ICU-ARDS and normal controls from tryptic or optional phospho/tryptic peptides showed that CYTB, ND5, MRPL37 and ALDH2 were the most specifically elevated mitochondrial proteins in COVID-19 plasma. Organizing the results from the plasma discovery and Chi Square analysis into a Venn Diagram showed that some mitochondrial components specific to viral infection were up-regulated in COVID-19 versus both ICU-ARDS and/or NHP samples (Fig. 3). Central members of the cytochrome system including CYTB and ND5 as well as mitochondrial ribosome components (MRPL) and central metabolic enzymes such as Aldehyde dehydrogenase (ALDH2) and L-2-Hydroxyglutarate dehydrogenase (L2HGDH) were increased in COVID-19 compared to normal human plasma and/or ICU-ARDS patients.

### Luminol oxidase activity

**Fig. 4:**
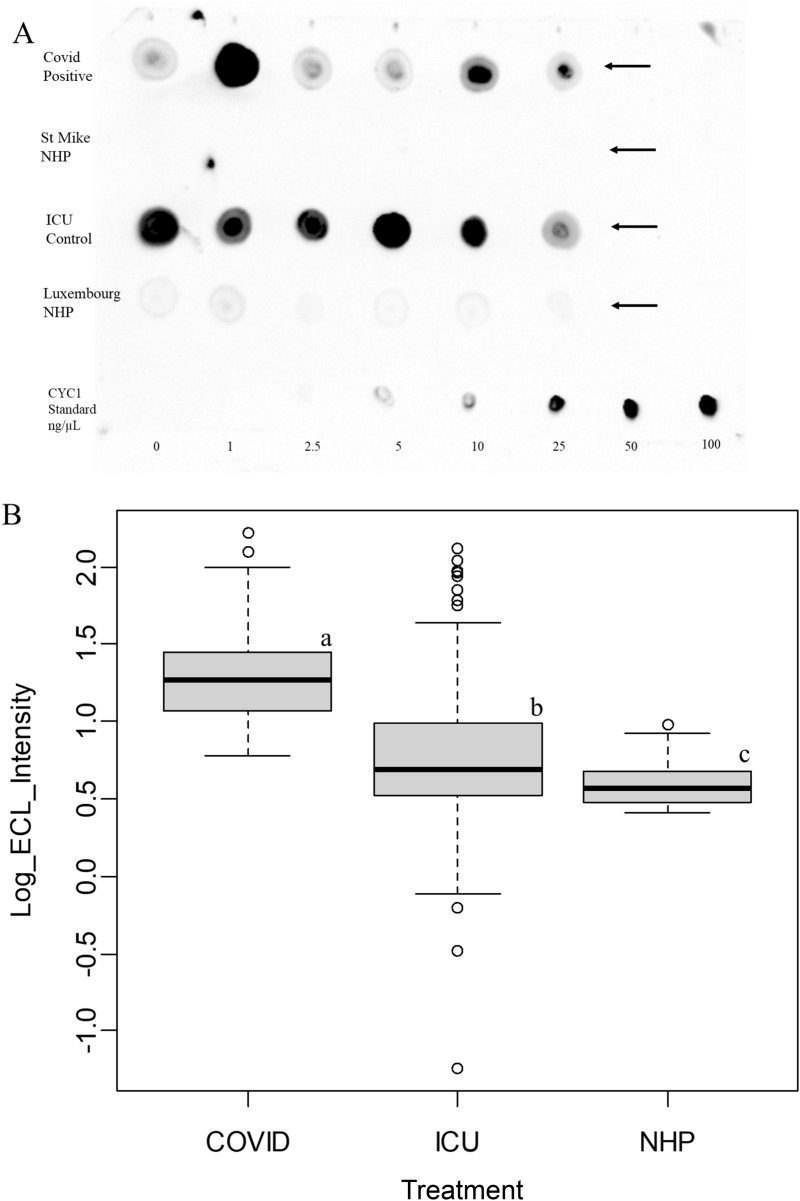
The presence of a luminol-oxidase activity in the plasma of COVID-19 and ICU Acute Respiratory Distress Syndrome patients (ICU-ARDS) compared to Normal Human Plasma (NHP). **A** One microlitre (1 µL) of a representative set of COVID-19 plasma was spotted onto PVDF alongside a specifically selected set of ICU-ARDS plasma (positive control) and two sets of representative NHP plasma (negative control) ECL dot blots. Cytochrome c from 0 to 100 ng/µL served as a detection standard. Arrows show the sample lanes; **B** Quantile boxplot of the intensity of luminol oxidase activity from the complete set of COVID-19, ICU-ARDS and NHP plasma samples in the study. The results from the two independent batches of normal that showed similar results were combined for graphic and statistical analysis. Significant difference by the Tukey Kramer Honestly Significant Difference (HSD) test at the p ≤ 0.05 level are shown by lower case letters

Proteins of the cytochrome electron transport chain of the mitochondria including MT-ND, NDUF, complex IV (COX), CYBR, CYC, CYB and CYTB, but also CYP, have all been previously observed to participate or contribute to luminol or lucigenin oxidation in vivo and/or in vitro [15, 22, 27, 29–32, 61, 62]. Similarly other heme containing oxidases or peroxidases have also been implicated in the response to infection [63]. Cytochrome and cytochrome oxidase enzymes have been previously shown to react with luminol or lucigenin in the presence of H_2_O_2_ to yield chemiluminescence in vitro [27, 29–32]. No signal was observed in the absence of H_2_O_2_ from the luminol test solution. Testing plasma for H_2_O_2_ (Hydrogen peroxide) dependent cytochrome-like activity using luminol (Fig. 4A) showed a significant increase in the ECL signal intensity in COVID-19 and ICU compared to Normal plasma (Fig. 4B). There was apparently the equivalent of ng/µL (µg/mL) levels of cytochrome c-like activity in COVID-19 and ICU-ARDS plasma while NHP was near the lowest detection levels.

## Discussion

The aim of this study was to orchestrate standard biochemistry techniques followed by LC-ESI-MS/MS with classical computation and statistical methods for primary discovery of plasma proteins from COVID-19 and ICU-ARDS versus normal controls. Traditional protein precipitation and standard partition chromatography were followed by tryptic digestion and LC–ESI–MS/MS. The resulting peptide identifications were analyzed by the classical statistical practice of random and independent sampling for Chi Square comparison of corrected peptide observation frequency. The type I error rate of protein identification with respect to random expectation was estimated using the Monte Carlo controls of computer random and noise random MS/MS spectra. Herein, the orchestration of well-established laboratory approaches and classical statistical methods has revealed the mitochondrial components and heme-containing oxidases such as cytochromes released from the cells of severe COVID-19 or ICU-ARDS patients that can be directly measured by a rapid and simple enzyme assay.

### Mitochondrial DNA PCR assay

The PCR amplification of mitochondrial ND1 was a sensitive method to detect mitochondrial components in COVID-19 or ICU-ARDS plasma and demonstrated that macromolecules from the mitochondria may be released from cells in agreement with previous results [19]. However, the PCR assay was so sensitive it detected mitochondrial DNA in many of the NHP control samples and so apparently did not show much discrimination in the detection of MT DNA.

### Analysis of human COVID-19 plasma proteins

The COVID-19 plasma proteome was recorded using manual plasma precipitation, isolation of proteins by disposable chromatography for tryptic digestion, manual collection and injection of peptides for random sampling by tandem mass spectrometry alongside random MS/MS spectra controls. The laborious manual procedure enabled the detection and quantification of cellular proteins that were released into the plasma in COVID patients. The significant difference between authentic sample observation frequency versus computer random and noise MS/MS spectra, and the significant difference between treatments by ANOVA and Chi Square, were consistent with an apparently low type I error rate of protein discovery from the fit of MS/MS spectra. The results of this study showed good agreement with previously reported analysis of COVID-19 plasma regarding common acute phase markers such as AZGP1 ORM, CRP, SAA1, HP, ORM, HPR and B2M that were increased in COVID-19 patients [1–11], that confirmed the sampling scheme, instrumental analysis and computations were successful. The reduction in apolipoproteins in COVID-19 was consistent with ELISA assays [64]. The sensitivity to the spectrum of acute phase markers and apolipoproteins is a clear demonstration of the efficacy of random and independent sampling by LC–ESI–MS/MS with frequency-based analysis using classical statistical methods in R alongside the Monte Carlo statistical controls of computer random and noise MS/MS spectra.

### Mitochondrial proteins in plasma

There were 652 previously established structural or functional interactions between the small subset of mitochondrial proteins observed in COVID-19 plasma that may indicate the mitochondrial proteins take the form of protein complexes in circulation [60]. The results of LC–ESI–MS/MS showed excellent agreement with the recent literature on the mitochondrial apoptosis pathway that is activated in response to viral infection [8, 14–18, 20, 21]. An increasing repertoire of cytochrome p450s (CYP) is now known to be targeted to the mitochondria, especially CYPs associated with sterol and steroid synthesis [65, 66]. The release of mitochondrial protein enzymes such as oxidase components MT-CO2 and CYB, CYTB or CYP into circulation were consistent with cellular damage from viral infection that might be quantified by enzyme activity to serve as a prognostic marker for the severity of COVID-19 infection [67]. Cytochrome enzymes such as MT-COX, CYB, CYTB, or CYP are known to react with luminol to provide an ECL signal in vitro [29–31, 62]. The increased presence of the cytochrome oxidase MT-COX2, cytochromes such as CYB, CYTB, or CYP or associated factors in the plasma of ARDS and COVID-19 patients as discovered by plasma proteomics was consistent with the measurements of increased cytochrome-like activity in the plasma of patients with respiratory distress [29–32].

### Biology of SARS-CoV-2

**Fig. 5:**
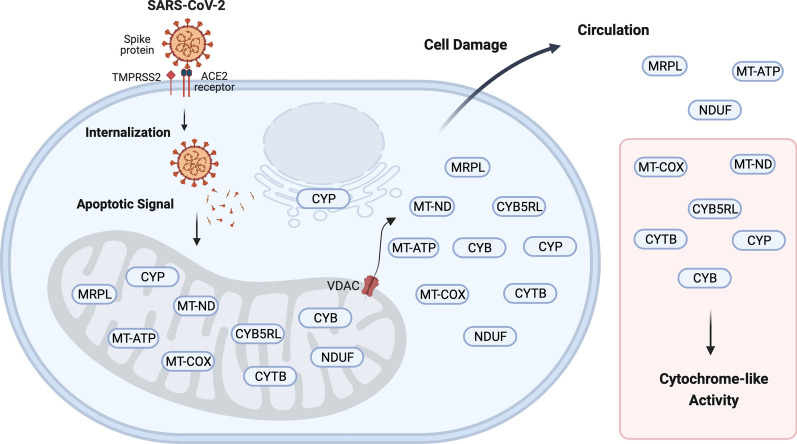
A cartoon summary of the mitochondrial proteins and enzyme activities discovered by LC–ESI–MS/MS from COVID-19 versus ICU and NHP sample populations. The SARS-CoV-2 spike protein is apparently cleaved by extracellular TMPRSS5 to gain entry via ACE2 and is unpacked in the cytosol releasing the spike and triggering expression of VDAC that releases the contents of the mitochondria including cytochrome, electron transport complex, ribosomal and others components such as MRPL, MT-ND, MT-COX, NDUF, CYB5RL and CYTB (see Table 4). Created with BioRender.com

Infection by the SARS-CoV-2 virus is known to result in the clustering of the mitochondria and the endoplasmic reticulum (ER) with the loss of normal compartments and integrity of the cells [8, 14–21, 68]. The SARS-CoV-2 spike protein may be cleaved by the protease TMPRSS2 to gain entry to the cell through the ACE2 receptors [13] (Fig. 5). Once inside the cell, the SARS-CoV-2 spike protein may act as a trigger of apoptosis via a mitochondrial pathway [14]. The increased expression of porins in the mitochondrial outer membrane in response to SARS-CoV-2 infection [8] provides a clear and established mechanism for the release of mitochondrial proteins from the organelle into the cytoplasm of cells infected with SARS-CoV-2 [18]. The porin VDAC may permit the loss of cytochrome enzymes from the mitochondria to the cytosol with eventual loss from the compromised cells [27, 29, 32, 61]. The release of cytochrome is a crucial part of the process that triggers cell death and apoptosis [15]. SARS-CoV-2 infection may lead to the fission of membrane-bound organelles and cellular lysis consistent with the escape of large molecules like mitochondrial components [8, 16, 20, 21, 28] including cytochromes and MT-COX proteins that might be detectable by luminol oxidase assays [29–32]. All of the data presented herein supports a mitochondrial pathway of apoptosis in SARS-CoV-2 infected cells that resulted in the release of mitochondrial proteins and DNA into the plasma that was similar to that observed in ICU-ARDS patients.

## Conclusion

The SARS-CoV-2 virus shows clear structural and functional associations with the mitochondria during viral infection and replication [17]. Four independent lines of evidence including the PCR against the oxidase component ND1, the increased observation frequency of mitochondrial proteins in plasma by LC–ESI–MS/MS, the previously established structural and functional interactions between the increased proteins and the presence of a cytochrome-like ECL activity in COVID-19 plasma were all consistent with the loss of mitochondria and cytochrome components from cells in severe SARS-CoV-2 infection. Here it was demonstrated for the first time that the plasma from clinical populations may be analyzed in the protein discovery laboratory by LC–ESI–MS/MS to reveal new cellular proteins and the results translated into a rapid and simple biochemical test for an enzyme activity in COVID-19 and ICU-ARDS patients.

## Abbreviations

ACN: Acetonitrile
ARDS: Acute respiratory distress syndrome
COVID-19: Infectious disease from SARS-CoV-2
ICU: Intensive care unit
LC–ESI–MS/MS: Nano liquid chromatography, electrospray ionization and tandem mass spectrometry
LIT: Linear ion trap
OIT: Orbital ion trap
NHP: Normal human plasma
STYP: Fully tryptic phosphopeptide
TRYP: Fully tryptic peptide
